# *Mycobacterium tuberculosis*: An Adaptable Pathogen Associated With Multiple Human Diseases

**DOI:** 10.3389/fcimb.2018.00158

**Published:** 2018-05-15

**Authors:** Qiyao Chai, Yong Zhang, Cui Hua Liu

**Affiliations:** ^1^CAS Key Laboratory of Pathogenic Microbiology and Immunology, Institute of Microbiology, Chinese Academy of Sciences, Beijing, China; ^2^Savaid Medical School, University of Chinese Academy of Sciences, Beijing, China

**Keywords:** *Mycobacterium tuberculosis*, pulmonary disease, autoimmune disease, metabolic disease, human microbiome

## Abstract

*Mycobacterium tuberculosis*, the etiological agent of tuberculosis (TB), is an extremely successful pathogen that adapts to survive within the host. During the latency phase of infection, *M. tuberculosis* employs a range of effector proteins to be cloud the host immune system and shapes its lifestyle to reside in granulomas, sophisticated, and organized structures of immune cells that are established by the host in response to persistent infection. While normally being restrained in immunocompetent hosts, *M. tuberculosis* within granulomas can cause the recrudescence of TB when host immunity is compromised. Aside from causing TB, accumulating evidence suggests that *M. tuberculosis* is also associated with multiple other human diseases, such as pulmonary complications, autoimmune diseases, and metabolic syndromes. Furthermore, it has been recently appreciated that *M. tuberculosis* infection can also reciprocally interact with the human microbiome, which has a strong link to immune balance and health. In this review, we highlight the adaptive survival of *M. tuberculosis* within the host and provide an overview for regulatory mechanisms underlying interactions between *M. tuberculosis* infection and multiple important human diseases. A better understanding of how *M. tuberculosis* regulates the host immune system to cause TB and reciprocally regulates other human diseases is critical for developing rational treatments to better control TB and help alleviate its associated comorbidities.

## Introduction

It has been over a century since Robert Koch identified the etiological agent of human tuberculosis (TB), termed *Mycobacterium tuberculosis*. However, to date, this pathogen continues to be a problem for human health. In 2016, there were still ~10.4 million TB cases, including 600,000 rifampicin-resistant TB (RR-TB) and 490,000 multidrug-resistant TB (MDR-TB) cases (WHO, 2017). Different from many other bacterial pathogens that secrete various toxins to cause acute inflammation and severe tissue damage (Ramachandran, 2014), *M. tuberculosis* is an extraordinary paradigm of intracellular pathogens that does not possess classical virulence factors. Indeed, it can persist in the host during long-term latency without causing significant damage or transmission unless the host immunity is compromised, e.g., when the host is treated with TNF-α blockers or co-infected by human immunodeficiency virus type-1 (HIV-1) (Shim, 2014; Bell and Noursadeghi, 2017). *M. tuberculosis* secretes a range of effector proteins to confuse the host immune system, thus promoting its intracellular survival and shaping its lifestyle to persist in granulomas during the latency phase of infection (Gröschel et al., 2016).

Aside from causing TB, increasing evidence suggests that *M. tuberculosis* is also associated with multiple other human diseases, such as pulmonary complications, autoimmune diseases, and metabolic syndromes (Table 1). Furthermore, *M. tuberculosis* infection can also reciprocally interact with the human microbiome, which has a strong link to immune balance and health conditions. In this review, we begin with a description of the adaptive survival of *M. tuberculosis* within the host. Then, we provide an overview for regulatory mechanisms underlying interactions between *M. tuberculosis* infection and multiple important human diseases. A better understanding of how *M. tuberculosis* regulates host cellular functions to cause TB and aggravate other human diseases under certain circumstances is critical for developing more rational strategies for TB control.

**Table 1:** Systematic studies of the links between TB and concurrent diseases.

**TB comorbidity**	**Number of studies included**	**Number of countries included**	**Year**	**Result**	**Reference**
COPD ^*^	19	27	1955-2011	A positive association between a past history of TB and the presence of chronic airflow obstruction.	Allwood et al., 2013
Lung cancer	42	9	1966-2009	A significant association between TB with adenocarcinoma.	Liang et al., 2009
Sarcoidosis	13	7	1990-2015	The existence of an association between *M. tuberculosis* and sarcoidosis.	Fang et al., 2016
SLE ^*^	6	4	2008	TB incidence was higher in the SLE group compared to the control.	Prabu and Agrawal, 2010
DM ^*^	13	8	1965-2007	DM was associated with higher TB risk in spite of study design and population	Jeon and Murray, 2008
Obesity	6	4	2008	A log-linear inverse relationship between TB incidence and body mass index.	Lönnroth et al., 2010
Hypovitaminosis D	7	8	1980-2006	Low serum vitamin D levels were associated with higher risk of active TB	Nnoaham and Clarke, 2008

* *COPD, chronic obstructive pulmonary disease; SLE, systemic lupus erythematosus; DM, diabetes mellitus*.

## Adaptive survival of *M. tuberculosis* in the host

*M. tuberculosis* is a highly adaptive pathogen living inside the host. For better insights into its role in human diseases, we first introduce the molecular mechanisms underlying the dynamic interactions between *M. tuberculosis* and the host.

### Host adaptation of *M. tuberculosis* with symbiotic features

*M. tuberculosis* can cause both pulmonary TB and extrapulmonary TB (EPTB) such as TB lymphadenitis, pleural TB, ocular TB, skeletal TB, and gastrointestinal TB (Shah and Chida, 2017). Thus, *M. tuberculosis* has adapted to various anatomic sites of the host body after eons of co-evolution with its host, and it exhibits some symbiotic features as follows:

First, *M. tuberculosis* can be regarded as a conditional pathogenic bacterium in a sense because it only causes TB in immunocompromised hosts. Over 90% of *M. tuberculosis*-infected individuals can spontaneously control the infection (Cambier et al., 2014). During the latency phase, *M. tuberculosis* establishes a commensal-like relationship with the host without causing obvious symptoms. Furthermore, certain *M. tuberculosis* antigens and the vaccine strain *Bacillus* Calmette-Guérin (BCG) have been shown to boost antitumor immune responses while being used in cancer therapy (Zbar et al., 1970; Hanna et al., 1972; Koyama et al., 2015, 2016; Zhang et al., 2015).

Second, similar to the features of symbiotic microorganisms privileged with host immune ignorance, *M. tuberculosis* is able to persist in the host by largely suppressing both innate and adaptive immunity (Goldberg et al., 2014). Indeed, even indigenous bacteria such as the intestinal microbiota can trigger host immune responses via Toll-like receptors (TLRs) in the intestinal epithelia. Normally, however, the mucosal immune system maintains ignorance by strictly confining the symbionts to the intestinal lumen. This is accomplished by mucin glycoproteins secreted from goblet cells that form a double mucus layer covering the epithelial surface (Hooper, 2009). Similarly, *M. tuberculosis* is commonly observed as tightly sequestered in nodule-like structures termed granulomas, which are believed to be able to restrict bacteria as long as host immunity remains sufficiently effective.

Third, *M. tuberculosis* in stable granulomas is always in a metabolically active but non-growing state termed “quiescence” (Rittershaus et al., 2013). Surprisingly, metabolic and genetic maps indicate that quiescent *M. tuberculosis* can balance its population by continuously adapting to the highly dynamic environment in the granulomas (Pienaar et al., 2016). Growing evidence indicates that *M. tuberculosis* possesses an elaborate gene regulatory network in response to external stimuli for acclimation to the host hypoxic environment (Forrellad et al., 2013; Galagan et al., 2013).

Fourth, *M. tuberculosis* employs multiple eukaryotic-like effectors to mimic or modify host signaling pathways and cellular functions. Eleven eukaryotic-like serine/threonine protein kinases (PknA to PknL) and two serine/threonine phosphatases (PtpA and PtpB) are encoded by *M. tuberculosis*, all of which are of vital importance for maintaining bacteria survival in the host (Forrellad et al., 2013). For instance, we previously identified a unique ubiquitin-interacting motif-like (UIML) domain in PtpA that imitates the eukaryotic function for binding ubiquitin, a small ubiquitous protein that participates in diverse host cell signaling pathways (Wang et al., 2015).

Despite the uncertainty of the commensal or symbiotic features of *M. tuberculosis*-host coexistence, it is anticipated that pathogen-host interacting interface-based adjunctive therapies will help minimize TB infection and avoid severe tissue damage by restoring host homeostasis.

### Granulomas: the symbol of balance

Once inhaled into the lungs through the trachea, *M. tuberculosis* is engulfed by alveolar macrophages (AMs) and captured into phagosomes, which subsequently deliver their cargoes to lysosomes for degradation (Pieters, 2008; Cambier et al., 2014). However, in many cases, *M. tuberculosis* can effectively block the acidification and maturation of phagosomes to survive in host AMs (Ehrt and Schnappinger, 2009; Houben et al., 2012). Even so, the invaded mycobacteria would be restricted by host forming granulomas with infected macrophages surrounded by layers of immune cells including granulocytes, dendritic cells (DCs), natural killer (NK) cells, and T and B lymphocytes. Actually, a considerable proportion of infected individuals are competent to clear the pathogen and develop sterile granulomas. On the other side, in order to overcome host immune responses and hypoxic environment, *M. tuberculosis* remains in a quiescent status within the granulomas in nearly 90% of the infected individuals (Rittershaus et al., 2013; Bhavanam et al., 2016). Both innate (e.g., secretion of IFNγ, TNFα, and other antimycobacterial factors from macrophages) and adaptive (e.g., Th17 cells, CD4^+^, and CD8^+^ T cells-mediated immunity) immune defenses are involved in keeping *M. tuberculosis* under control during the latent phase of TB infection (Dutta and Karakousis, 2014). However, in 5–15% of the infected cases, *M. tuberculosis* can be reactivated to replicate (WHO, 2017). For example, when the host immunity is compromised, *M. tuberculosis* becomes activated to start replication, which leads to necrosis of infected macrophages and the release of the intracellular bacteria, which could further infect new cells and spread to other tissues (Dutta and Karakousis, 2014).

**Figure 1:**
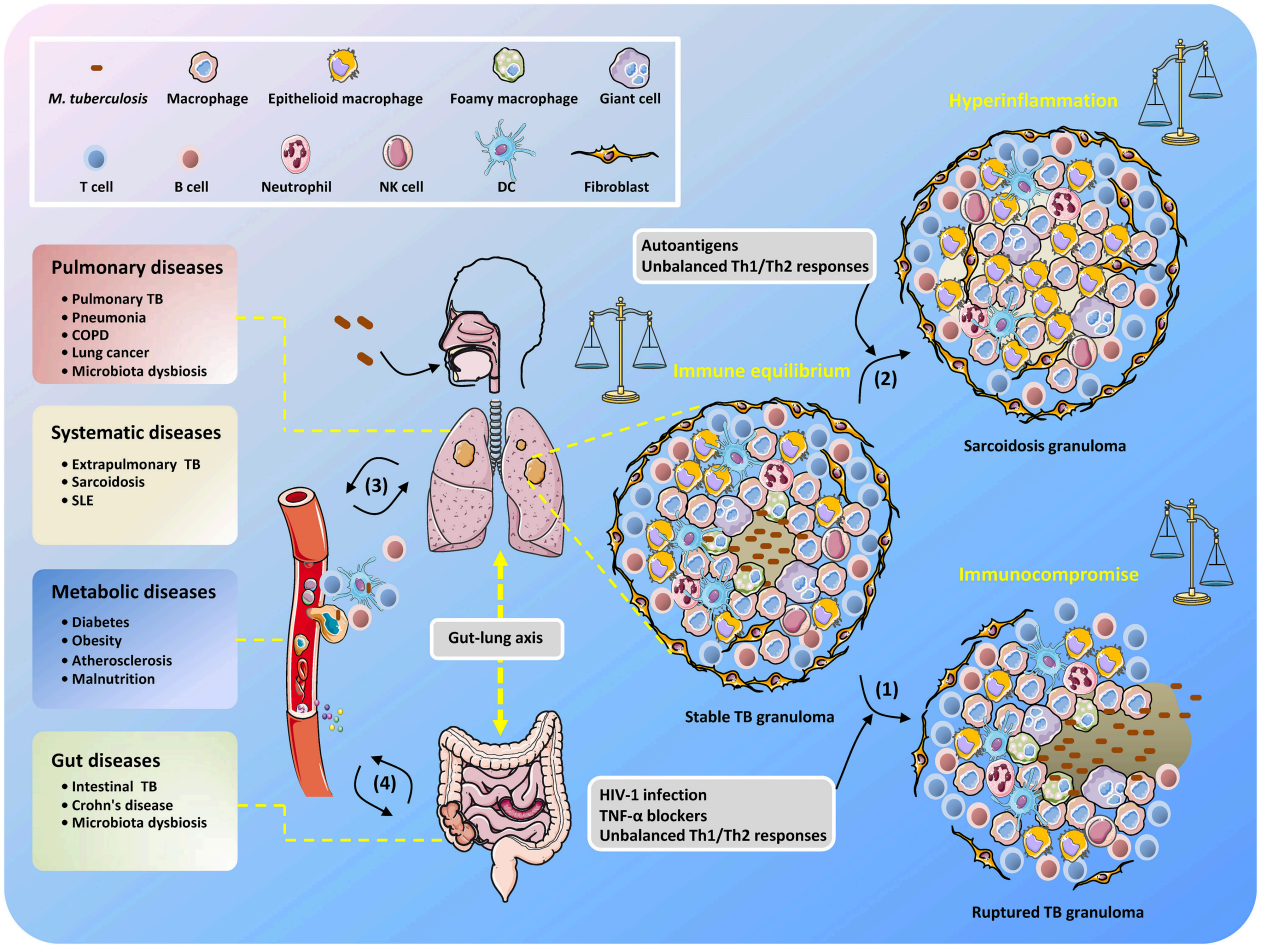
Unbalanced immune system in TB patients results in the development of diverse diseases. After inhalation of *M. tuberculosis*, granulomas are formed with piles of immune cells to sequester the uncleared bacteria that subsequently step into latency. When the host becomes immunocompromised, *M. tuberculosis* is reactivated to replicate and disseminate, which is accompanied by granuloma caseating, liquefying, and cavitating (1). Non-caseating granulomas may continously exist after bacteria elimination and present as sarcoidosis because of excessive host inflammatory immune responses (2). The infected cells, *M. tuberculosis* conponents, metabolites, and host immune molecules such as cytokines and chemokines are able to be exchanged and transmitted via the circulatory system, thus increasing the risk of disease development (3). The gut microbiota is also involved in the interplay between *M. tuberculosis* and TB comorbidity via the gut-lung axis (4).

Thus, maintaining solid granulomas in infected individuals is a hallmark of the balanced confrontation between *M. tuberculosis* and the host, and any disequilibrium may cause disease development (Figure 1). Immune homeostasis in granulomas can be regulated by AMs with two polarized types, known as classically activated macrophages (M1 macrophages) and alternatively activated macrophages (M2 macrophages) (Hussell and Bell, 2014). M1 macrophages are responsive to Th1-dominant immunity stimulated by IFN-γ and TNF and can efficiently produce antibacterial agents like reactive oxygen species (ROS) and nitric oxide (NO), as well as pro-inflammatory cytokines (e.g., TNF, IL-1β, and IL-6) (Gordon, 2003). By contrast, differentiation into M2 macrophages is trigged by cytokines IL-4 and IL-13 produced by Th2 cells (for M2a macrophages), immune complexes and pattern recognition receptors (PRRs) (for M2b macrophages), and IL-10 produced from T regulatory (Treg) cells (for M2c macrophages) (Mantovani et al., 2004). M1 macrophages are crucial for suppressing TB progression, but uncontrollable inflammation may induce severe tissue damage (Hussell and Bell, 2014), which can be counterbalanced by M2 macrophage- and Th2 cell-mediated immunosuppressive regulation. Remarkably, it seems that during TB development, the host is biases to Th2-type immunity, which is deemed to be beneficial for the intracellular survival of *M. tuberculosis* (Raju et al., 2008; Schreiber et al., 2009; Redente et al., 2010). Further elucidation of the regulatory mechanisms that control the balance of Th1/Th2 immune responses in *M. tuberculosis*-containing granulomas will undoubtedly be an exciting field of research in the coming years, and such studies could provide important knowledge for the effective manipulation of Th1/Th2 balance for the control of TB infection while minimizing associated host tissue damage.

### Invasion of *M. tuberculosis* into targeted cells

Surprisingly, recent studies suggest that mycobacteria are able to invade various non-canonical immune cells, including epithelial cells, endothelial cells, fibroblasts, adipocytes, and neuronal cells (Randall et al., 2015). This ability of *M. tuberculosis* may explain why TB infections can appear at any anatomic site and disseminate to multiple organs.

A key prerequisite for intracellular colonization of mycobacteria is their ability to adhere and enter host cells. To date, an array of host PRRs has been identified that mediate phagocytosis of *M. tuberculosis*. Those PRRs include mannose receptors, complement receptors, Fc receptors, and C-type lectin receptors, including dendritic cell-specific intercellular adhesion molecule-3-grabbing non-integrin (DC-SIGN) and macrophage inducible C-type lectin (Mincle) (Ernst, 1998; Ishikawa et al., 2009). In contrast, few molecular components from *M. tuberculosis* have been identified to facilitate this event with clear mechanisms. Heparin-binding hemagglutinin adhesin (HBHA) was the first defined adhesin in *M. tuberculosis* that is crucial for extrapulmonary dissemination of *M. tuberculosis* (Menozzi et al., 1998; Pethe et al., 2001). Loss of HBHA decreases mycobacterial adhesion and the invasion of epithelial cells but not macrophages (Pethe et al., 2001). Recently, *M. tuberculosis* pili (MTP) have also been suggested to function as important adhesion molecules affecting mycobacteria-host cell interactions (Ramsugit et al., 2016). Mammalian cell entry (Mce) family proteins are another group of bacterial surface-exposed molecules associated with host cell entrance. The *mce1* gene recombined into *Escherichia coli* confers the bacteria with the capacity to invade epithelial cells (Arruda et al., 1993). Lately, our lab has confirmed that Mce3C can also facilitate the internalization of *M. tuberculosis* into macrophages by exploiting the β2 integrin-mediated signaling pathway (Zhang Y. et al., 2017).

### Intracellular survival of *M. tuberculosis* with counterbalanced host immune defenses

The host exerts both innate and adaptive immune functions to protect against *M. tuberculosis* infection. Initially, the innate immune cells rapidly respond by direct recognition of conserved pathogen-associated molecular patterns (PAMPs) like lipoproteins, glycolipids, and carbohydrates on the *M. tuberculosis* cell surface (Killick et al., 2013). Subsequently, a variety of immune mechanisms, such as phagocytosis, autophagy, apoptosis, and inflammasome assembly, are evoked to efficiently control *M. tuberculosis* survival (Liu et al., 2017). Finally, adaptive immunity such as Th1/Th17 responses mediated by *M. tuberculosis*-specific CD4^+^ T cells become involved, which plays a pivotal role in the control of TB progression (Jasenosky et al., 2015). In addition, recent studies imply that B cell-mediated humoral immunity can also manipulate the inflammatory responses in TB granulomas to control local infection, despite a minor effect on overall pathology or disease progression being observed (Maglione et al., 2007; Kozakiewicz et al., 2013; Phuah et al., 2016).

Notably, host adaptive immunity to *M. tuberculosis* is activated after a considerably longer interval compared to other pathogen infections. Several studies demonstrate that the CD4^+^ T cell response does not initiate until 10–14 days after infection and peaks at nearly 3 weeks after infection by *M. tuberculosis* in mice (Chackerian et al., 2002; Khader et al., 2007; Wolf et al., 2008). This delayed response has not yet been entirely explained, though one possibility is that *M. tuberculosis* may suppress the function of DCs and limit their migration from the lungs to lymph nodes for activation of initial T cells (Wolf et al., 2008; Divangahi et al., 2010; Roberts and Robinson, 2014; Griffiths et al., 2016).

Host innate immunity against *M. tuberculosis* has gained increasing attention in recent years because the innate immune cells are at the frontline of the defense against infection and also the main effector cells after being further activated by adaptive immunity. Upon infection by *M. tuberculosis*, the host orchestrates multiple signaling cascades via the PRRs to launch a variety of innate immune defense functions, which are subverted by secreted *M. tuberculosis* effector proteins. *M. tuberculosis* delivers effector proteins via 6 kDa early secretory antigenic target (ESAT6) protein family secretion (ESX) systems (classified as type VII secretion systems), including ESX-1 to ESX-5 (Gröschel et al., 2016). The ESX-1 system is the most studied secretion system in mycobacteria and has been well-characterized for its vital effects on host immune triggering (e.g., activation of the Type I IFN response and NLRP3/ASC inflammasome) or evasion (e.g., phagosomal rupture and subversion of autophagic flux) (Stanley et al., 2007; Mishra et al., 2010; Romagnoli et al., 2012; Simeone et al., 2012). Notably, ESX-1 is not the exclusive system that determines mycobacterial pathogenesis, because some effector proteins expressed in BCG, which spontaneously lacks the *esx-1* locus, are still secreted and effective in facilitating bacterial survival in host cells. For example, we previously revealed that *M. tuberculosis* effector proteins such as PtpA and Mce3E modulate host innate immunity to promote the intracellular survival of mycobacteria (Li et al., 2015; Wang et al., 2015). Besides, an increasing number of studies indicate that the ESX-5 system, which exports an array of mycobacterial PE and PPE family proteins to modulate host immunity, is another secretion machinery that plays a critical role in mycobacteria-host interactions (Chen and Xie, 2010; Gröschel et al., 2016). Interestingly, host innate immune cells also apply restriction factors to antagonize the function of pathogen effector proteins. For example, many tripartite motif (TRIM) family proteins have been demonstrated to serve as host restriction factors via regulating innate immunity and counteracting the effects of some viral effector proteins (Hatakeyama, 2017). In the case of *M. tuberculosis*, our lab identified the host protein tripartite motif containing 27 (TRIM27) as a potential host restriction factor that can directly interact with *M. tuberculosis* PtpA and control bacterial survival in macrophages by enhancing inflammatory responses and cell apoptosis (Wang J. et al., 2016). Thus, there exists an evolutionary dynamics of interactions between host restriction factors and pathogen antagonists during mycobacterial infection.

## *M. tuberculosis* and pulmonary diseases

As the portal of bacterial entry, the lung is the most commonly affected organ during *M. tuberculosis* infection. The respiratory tract and the bronchoalveolar spaces represent a unique immunological compartment where diverse tissue-specific cells shape the first-line immune response to inhaled *M. tuberculosis*. These interactions alter the pulmonary microenvironment, which may remodel the airways and influence the development and outcomes of various pulmonary diseases.

### Chronic obstructive pulmonary disease (COPD)

COPD is characterized by airflow limitation associated with an enhanced chronic inflammatory response, both in the airway and the lung, to noxious particles or gases, such as the substances produced by tobacco smoking. Recently, epidemiological studies have provided a strong link between past history of TB and later development of chronic airflow obstruction (Amaral et al., 2015; Byrne et al., 2015). Growing evidence argues that pulmonary TB can lead to remodeling of the lung architecture, which can be manifested as extensive fibrosis, cavitation, traction bronchiectasis, bronchostenosis, or parenchymal lung destruction (Dheda et al., 2005; Jordan et al., 2010). This may be a possible explanation for the development of COPD in patients with a previous history of TB. Chronic airflow obstruction is also suggested to be a sequel to active TB because of the development of bronchiectasis in patients with COPD (Chakrabarti et al., 2007). A common link to the pathogenesis of both conditions may lie in the destruction of the pulmonary extra-cellular matrix (ECM), which might be caused by various risk factors, such as smoking and biomass fuel exposure (Elkington and Friedland, 2006; Løkke et al., 2006). In addition, human pneumonia, as another pulmonary disease that commonly occurs in TB patients (especially adolescents who have strong immunological responses), can also increase morbidity and mortality rates related to acute exacerbations of COPD (Modesto dos Santos et al., 2014; Oliwa et al., 2015).

### Lung cancer

Both TB and lung cancer represent global threats claiming millions of lives worldwide. Clinical data show that TB and lung cancer may co-exist in some cases, and the history of TB infection is reported as a risk factor for lung cancer development (Brenner et al., 2001; Engels et al., 2009). As has been described, *M. tuberculosis* can escape host immune defenses and establish chronic and persistent inflammation. Chronic inflammatory conditions are thought to create the appropriate microenvironment for cancer development by a number of mechanisms. Specifically, the higher rate of cell turnover likely increases the risk for genetic errors to trigger carcinogenesis. For instance, various mycobacterial cell wall components may induce production of NO and ROS to cause DNA damage (Sharma et al., 2004; Shin et al., 2008), which is implicated in inflammation-related carcinogenesis (Kawanishi et al., 2006). Additionally, the enhanced cell-mediated immune responses caused by mycobacterial infection could result in the extension of pulmonary fibrosis, which could probably contribute to the development of lung scar carcinoma (Ardies, 2003). Intriguingly, recent research from our laboratory have demonstrated that *M. tuberculosis* PtpA, which is a secreted effector protein that enters into the nucleus of host cells, could promote proliferation and migration of human lung adenoma A549 cells *in vitro* and in a mouse xenograft model (Wang J. et al., 2017). Animal experimental evidence also revealed that chronic TB infection could lead to cell dysplasia and squamous cell carcinoma specifically in the lung (Nalbandian et al., 2009). Because the causal relationship between TB and lung cancer remains inconclusive and similarities between those two lung diseases might mislead diagnosis, further investigations are needed to fully understand the underlying mechanisms linking *M. tuberculosis* infection to lung cancer development.

## *M. tuberculosis* and autoimmune diseases (AIDs)

The development of AIDs is determined by a combination of genetic, hormonal, and environmental factors, as well as the immunological status of the individual. Mycobacterial infection is implicated as an environmental trigger to initiate AIDs. For example, intravesical administration of BCG for bladder cancer triggers systemic autoimmunity (Sampaio et al., 2017). Clinical data also support the notion that *M. tuberculosis* may be involved in autoimmunity. For example, autoantibodies associated with AIDs, such as Wegener's granulomatosis and systemic lupus erythematosus (SLE), are detected in 40% of TB patients (Kakumanu et al., 2008).

There are three possible mechanisms accounting for the potential development of AIDs following TB infection. The first mechanism is molecular mimicry by which the mycobacterial components incorporate an epitope that is structurally similar to that of a self-antigen (Blank et al., 2007). For example, *M. tuberculosis* heat shock protein 60 (HSP60) and HSP65 are autoantigens present in the sera of patients with AIDs (Ribeiro et al., 2010). The second mechanism is bystander activation, a state where enhanced cytokine production, such as IFN-γ and TNF-α, induces the expansion of autoreactive T cells (Gilbertson et al., 2004; Shoenfeld et al., 2008). Third, TLR-mediated signaling activation caused by *M. tuberculosis* infection may also be involved in the pathogenesis of AIDs (Leadbetter et al., 2002; Rifkin et al., 2005).

Next, we discuss two TB-associated AIDs, sarcoidosis and SLE, with an aim to better elucidate the intricate relationship between mycobacterial infection and autoimmunity.

### Sarcoidosis

Sarcoidosis, an AID with no definite proof of an infectious etiology, is characterized by the presence of non-caseating granulomas with accumulated epithelioid cells in various organs. A previous study reports that mycobacterial DNA can be identified in sarcoidosis lesions (Song et al., 2005), suggesting that mycobacteria are either the cause or at least an important cofactor in the pathogenesis of sarcoidosis. Furthermore, mycobacterial HSPs are detected with high expression in sarcoidosis tissue as well, suggesting that those proteins may also participate in the etiopathogenesis of sarcoidosis. *M. tuberculosis* HSP16 and HSP70 are associated with early and later stage sarcoidosis, respectively (Dubaniewicz et al., 2006). Remarkably, multiple effector proteins secreted by *M. tuberculosis* can serve as agonists for TLRs to regulate the release of cytokines, thereby affecting the immunological events involved in lesion formation of lung sarcoidosis. For example, bronchoalveolar lavage cells from sarcoidosis patients exhibit increased cytokine responses to the 19-kDa lipoprotein of *M. tuberculosis* (LpqH), a TLR2/1 ligand (Gabrilovich et al., 2013). Due to the similar clinical and histopathological features of sarcoidosis and TB, some investigators argue that they may be the same disease with varying presentations (Agrawal et al., 2016; Elkington et al., 2016). However, noting that anti-TB treatment seemly has little effect on sarcoidosis patients (Dubaniewicz et al., 2013), more research and clinical trials are warranted to better elucidate this potential link.

### SLE

SLE is a systemic disease of unknown etiology that is characterized by autoantibodies against self-antigens, resulting in various inflammation-mediated systemic symptoms. Infections, renal failure, and cardiovascular diseases account for the majority of deaths seen in SLE patients (Ward et al., 1995). Several studies suggest that patients with SLE are at increased risk of reactivation and dissemination of TB due to multiple immune abnormalities and immunosuppressive therapy (Yun et al., 2002). Meanwhile, there is also growing evidence that supports the pivotal role of mycobacterial infection in induction and exacerbation of SLE (Ghosh et al., 2009). In this context, several studies report the development of SLE after TB infection and the presence of autoantibodies, such as antinuclear antibodies (ANA) and rheumatoid factor (RF), in the sera of patients with active TB are attributed to cross-reactivity between mycobacterial and host self-antigens (Amital-Teplizki et al., 1989). Additionally, several studies indicate that host vitamin D and TLR signaling cascades may also partially explain the association between TB and SLE (Papadimitraki et al., 2007; Nnoaham and Clarke, 2008). Taken together, it can be postulated that *M. tuberculosis* could be an immunomodulatory agent that precipitates the development of SLE, but a more comprehensive understanding of the common signaling pathways and crucial nodules involved in these two diseases remain to be defined.

## *M. tuberculosis* and metabolic syndromes

The metabolic system plays a central role in maintaining the stable nutritional status of the host, which is crucial for anti-TB immunity (Paranandi and Wanke, 2017). The effect of host metabolism on *M. tuberculosis* infection is primarily due to the bioenergetic demands for effective immune activation. Sensing pathogenic threats by innate immune cells triggers the responses of immune signaling networks, allowing a bulk of genes to initiate transcription and expression. This is followed by the release of inflammatory cytokines and chemokines and, subsequently, the expansion and differentiation of adaptive immune cells. Undoubtedly, sustaining the efficiency of these immune processes is faced with the urgent need for energy, resulting in a metabolic switch from oxidative phosphorylation to aerobic glycolysis (also known as the Warburg effect) (Warburg et al., 1958). Despite oxygen availability, the activated immune cells predominantly metabolize glucose into the pentose phosphate pathway (PPP) and prefer to excrete pyruvate generated by glycolysis as lactate rather than shuttle it into the tricarboxylic acid (TCA) cycle (Pearce and Pearce, 2013). NADPH, which is chiefly provided through the PPP, is required for the generation of NADPH oxidase-mediated ROS and thus contributes to the bactericidal efficacy of phagocytes. Furthermore, adaptive immune cells can also flexibly utilize other metabolites, such as glutamine via glutaminolysis, to supply the TCA cycle and fuel mitochondrial oxidative phosphorylation (OXPHOS) for their proliferation (Wang et al., 2011; Le et al., 2012). It should also be pointed out that metabolic reprogramming commonly occurs in both innate and adaptive immune cells (including M1 macrophages, neutrophils, DCs, NK cells, T, and B cells) after being activated (Gaber et al., 2017). A well-established paradigm is the metabolic disposition between M1 and M2 macrophages. Aside from the prominent activity of glycolysis and glutaminolysis, inducing M1 macrophages with TLR4 agonist also leads to a boost in the eicosanoid synthesis pathway, a metabolic process of polyunsaturated fatty acids that produces several inflammation-related metabolites like prostaglandins and leukotrienes (Norris et al., 2011; Dennis and Norris, 2015). By comparison, M2 macrophages induced by IL-4 are detected with little glycolytic activity and low flux via the PPP but, rather, with vigorous fatty acid oxidation (FAO) that fuels the TCA cycle and OXPHOS to supply energy (Vats et al., 2006).

It is increasingly appreciated that host metabolic disorders could benefit TB development, and *M. tuberculosis* tends to subvert host immune defenses by interfering with the metabolic system. Next, we summarize current knowledge on the reciprocal regulation of *M. tuberculosis* infection and several metabolic syndromes such as diabetes, obesity, atherosclerosis, and hypovitaminosis.

### Diabetes

Diabetes mellitus (DM), particularly type 2 diabetes (T2DM), is one of the strongest risk factors for TB disease that contributed to ~7.7% (~0.8 million incident cases) of total estimated TB cases in 2016 (WHO, 2017). In addition, cohort studies and clinical evidence suggest that people with DM have a nearly three-fold risk of active TB as compared to non-DM cases and may develop more severe symptoms in the lung (Jeon and Murray, 2008; Leung et al., 2017). Mounting studies on TB-DM comorbidity also provide evidence supporting the adverse impacts of DM on host anti-TB immunity. For example, several studies demonstrate that either spontaneous or chemically induced T2DM animal models (including mice, rats, and guinea pigs) infected with *M. tuberculosis* exhibit higher bacterial burden and more severe pathology than controls without DM (Sugawara et al., 2004; Martens et al., 2007; Podell et al., 2014). Studies also demonstrate that active TB patients with DM have elevated CD4^+^ Th1 and Th17 responses but reduced frequencies of Treg cells compared to patients without DM (Kumar et al., 2013), while DM patients with latent TB inversely have diminished Th1 and Th17 responses relative to patients without DM (Kumar et al., 2014). Another intriguing finding is that a strengthened host proinflammatory response and intensified mycobacterial proliferation only occur in chronic but not acute DM-TB infection models (Martens et al., 2007). Consistently, TB progression in DM models is not obviously different from that in normal controls until the late stage of infection (Podell et al., 2014). Taken together, these studies suggest that the impaired anti-TB immunity in DM patients may result from a long-term impact of disordered metabolic status that results from both DM and TB. However, the detailed mechanisms regarding how the courses of TB and DM influence each other through regulation of the metabolic system still remain largely unknown.

### Obesity

Obesity is answerable to the increasing global DM prevalence (Popkin, 2015). Interestingly, several studies suggest that overweight and obese individuals are conferred with protection against TB risk (Leung et al., 2007; Yen et al., 2017; Zhang H. et al., 2017). These results seem to be consistent with the findings that malnutrition exacerbates the morbidity and mortality of TB (Paranandi and Wanke, 2017). However, it is difficult to conclude that gaining weight is good for host anti-TB immunity because multiple confounding factors could be involved in these studies (e.g., different lifestyles and habits could exist between obese individuals and healthy ones). However, evidence based on animal experiments implies an immunomodulatory function of adipose tissue during *M. tuberculosis* infection. Leptin, an adipokine mainly secreted by adipocytes, is a pleiotropic factor involved in the metabolic, neuroendocrine, and immune systems (Abella et al., 2017). It has been shown that Leptin-deficient animal models exhibit a much weaker immune defense against mycobacterial infection (Wieland et al., 2005; Ordway et al., 2008). Furthermore, a lipidomics study in high-fat diet-induced obese mice revealed a distinct change of eicosanoid metabolites (Wang W. et al., 2016), which play important regulatory roles in adaptive immunity to *M. tuberculosis* infection (Divangahi et al., 2010). Nevertheless, further studies are warranted to better elucidate the impacts of obesity with altered metabolic status, such as elevated levels of fatty acids and cholesterol, on *M. tuberculosis* infection because host lipids are the major carbon sources for intracellular survival of *M. tuberculosis* (Nazarova et al., 2017).

### Atherosclerosis

Epidemic data imply a presumptive connection between TB and atherosclerotic disease (Chung et al., 2014; Wang S. H. et al., 2017). Consistently, mycobacterial HSP65 and the human homolog HSP60 are detected at heightened serum levels in atherosclerotic patients (Xu et al., 1993; Zhu et al., 2001). In addition, high-cholesterol diet-induced atherosclerotic animals immunized with HSP65 display an accelerated progression of disease, while oral tolerization with HSP65 can block the advanced formation of atherosclerotic plaques (Zhang et al., 2012; Wick et al., 2017). Atheroma has long been considered as a chronic infectious disease associated with multiple pathogens, including *M. tuberculosis, Chlamydia pneumoniae, Helicobacter pylori*, and periodontal bacteria (Anestad et al., 2001; Huaman et al., 2015). One possible explanation for this phenomenon could be that pathogen infection evoking a host Th1-type immune response may also intensify the inflammation at an early stage of plaque formation (Frostegard et al., 1999).

Interestingly, the immunological progression of atherosclerosis shares similar patterns with the formation of TB granulomas. Atherosclerosis is initiated by aberrant retention of oxidized low-density lipoprotein (oxLDL) in the arterial intima, which recruits inflammatory cells, including lymphocytes, monocytes, and macrophages, to the subendothelial space (Chinetti-Gbaguidi et al., 2015). Subsequently, the macrophages scavenge the lipoprotein particles by storing them as lipid droplets (LDs) in their cytoplasm and turn into foam cells. Increased deposition of oxLDL and inflammation result in apoptosis of these macrophages, leading to the formation of a necrotic core in the plaque, followed by further inflammation and thrombosis (Chinetti-Gbaguidi et al., 2015). Likewise, there are also foamy macrophages (FMs) distributed in the interface region around the necrotic center of TB granulomas (Peyron et al., 2008). These FMs containing quiescent *M. tuberculosis* are characterized by accumulated LDs, and they can translocate into the mycobacteria-containing vacuoles and potentially be hijacked by bacilli as energy reservoirs (Peyron et al., 2008; Barisch and Soldati, 2017). During chronic atherosclerosis, senescent FMs are deleterious throughout disease development, but due to the Th1-dominant response in the lesions, such FMs cannot be promptly cleared by M2 macrophages via efferocytosis (Viola and Soehnlein, 2015; Childs et al., 2016). Thus, it is intriguing to investigate the role of *M. tuberculosis* in manipulation of FMs in atherosclerotic plaques because *M. tuberculosis* seems to be able to assimilate the cytoplasmic LDs and might alter the M1/M2 polarization of macrophages at atherosclerotic plaques. Interestingly, one study has demonstrated a potential anti-atherosclerotic effect of BCG (van Dam et al., 2016), but further studies are needed to confirm this observation and to better understand the underlying mechanisms.

### Hypovitaminosis

Vitamins are essential micronutrients for maintaining individual health. One of the most studied vitamins is vitamin D, which demonstrates potential benefits in the treatment of multiple diseases, including TB (Feldman et al., 2014). Accumulating studies provide a mechanistic understanding of the effects of vitamin D signaling on the regulation of the host immune defense against *M. tuberculosis*. First, the activation of TLRs in mycobacteria-infected monocytes is indispensable to boost the synthesis pathway of 1,25-dihydroxy-vitamin D_3_ (1,25(OH)_2_D_3_), the active form of provitamin D_3_, which leads to the production of an antimicrobial peptide to kill the bacilli (Liu et al., 2006). Second, 1,25(OH)_2_D_3_ stimulates autophagy and phagosomal maturation, leading to the repression of mycobacterial growth and HIV-1 replication during co-infection (Campbell and Spector, 2012). Third, treatment of infected macrophages with vitamin D reduces the formation of lipid drops in the cytoplasm, which may limit the nutrition source for *M. tuberculosis* (Salamon et al., 2014). Accumulating data suggest that the level of serum vitamin D is decreased in TB patients but whether this decline is a causal risk factor of TB or a subsequent result of TB infection is undetermined (Azam et al., 2016; Wang Q. et al., 2017). Though it seems promising, the feasibility of utilizing micronutrients (such as vitamins D, C, and B6) with potential anti-TB effects as auxiliary anti-TB treatments still needs further confirmation (Dick et al., 2010; Vilchèze et al., 2013).

## Interplay of *M. tuberculosis* and the human microbiome

TB patients frequently suffer from co-infection with other pathogens such as HIV-1, *Helicobacter pylori* (Perry et al., 2010), and helminths (Babu and Nutman, 2016), which has led to the assumption that *M. tuberculosis* might have a complicated interplay with either exogenous pathogens or indigenous microbes. The Human Microbiome Project (HMP) has generated massive information on the composition of the human microbiome (Huttenhower et al., 2012; Lloyd-Price et al., 2017). Increasing evidence shows that the diversity of microbiota, which constitutes the integrated microbiome, can vastly vary in different niches within and among healthy individuals and has a strong influence on immune balance and health conditions. Here, we mainly focus on the description of the gut and lung microbiomes and the mutual regulation of these local microbial communities and *M. tuberculosis*.

### The gut microbiome

The human gut harbors nearly 100 trillion microbial cells and more than 160 bacterial species in each individual (Qin et al., 2010). The integration of these microbial communities has been proposed as a novel endocrine organ in humans (Clarke et al., 2014). By virtue of metabolites delivered from commensal microbes, the host immune system is substantially regulated by gut microbiota. For instance, bacterial short-chain fatty acids are abundantly found in the colonic lumen, among which, n-butyrate has been identified as an inhibitor of histone deacetylases in lamina propria macrophages to diminish cell proinflammatory responses (Chang et al., 2014). This hyporesponsiveness may benefit the immune ignorance of indigenous microbes. Another example is that tryptophan (Trp) metabolites secreted by gastrointestinal microbiota participate in host immunomodulation as revealed by recent work showing that indole-3-aldehyde produced by lactobacilli stimulates IL-22 transcription in innate lymphoid cells (ILCs) via the Trp metabolic pathway and promotes mucosal resistance against fungus (Zelante et al., 2013). Furthermore, a special subset of microbes can also colonize intestinal lymphoid tissues and reside in DCs to directly modulate cytokine production, which then boosts local Th17 and ILCs responses (Fung et al., 2016).

Nearly 20% of patients with gastrointestinal TB concomitantly have pulmonary TB (Shah and Chida, 2017). However, the gastrointestinal TB could be underreported because of limited and insensitive diagnostic approaches (Horvath and Whelan, 1998; Shah and Chida, 2017). The invasion of *M. tuberculosis* to the intestine is probably contributed to swallowing of contaminated food, hematogenous route from active pulmonary TB, and contiguous spread from adjacent infected organs (Horvath and Whelan, 1998). *M. tuberculosis* infection is assumed to trigger the dysbiosis of the microbiome and may be involved in gastrointestinal diseases. A study monitoring the dynamics of gut microbiota in mice before and after aerosol challenge with *M. tuberculosis* observed a continuous change in the community structure of the gut microbiota of the infected mice (Winglee et al., 2014). However, whether this disturbance of microbiota is a potential risk factor for developing other diseases remains unknown. Regardless, dysbiosis of the gut microbiota by antibiotic treatment in mice does promote the colonization and dissemination of *M. tuberculosis*, as well as skews the balance of Treg and Th1 cells (Khan et al., 2016). Intriguingly, an increasing number of studies reveal that *Mycobacterium avium* subspecies *paratuberculosis*, another pathogenic mycobacterium, acts as an infectious factor for inflammatory bowel diseases (IBDs) and has been isolated from the terminal ileum of patients with Crohn's disease (Chiodini et al., 1984; Naser et al., 2014). Because it is difficult to distinguish intestinal TB from an IBD such as Crohn's disease (Debi et al., 2014), it is attractive to compare the microbiome structures of intestinal TB and IBD to help reveal common regulatory mechanisms underlying both diseases and to identify biomarkers for differential diagnosis.

### The lung microbiome

The human respiratory microbiome remains a largely unexplored frontier. Only ~3% of publications have focused on the airway and lung biomes according to a PubMed-based search (Lloyd-Price et al., 2016). However, sequencing-based metagenomics and more recently developed microbial culturomics based on mass spectrometry techniques have complementarily provided access to the study of the non-cultivable species (Lagier et al., 2012), and further revealed a more comprehensive diversity of human microbiome including that in the respiratory tract. Compared to the human gut, the lung surface is topologically exposed to the outside environment with bidirectional air flow and microbial migration. Notably, microbes from the upper respiratory tract may immigrate into the lung by microaspiration because the bacterial communities in the oral cavity share similar membership with those in healthy lungs (Bassis et al., 2015). Any destabilization of the lung environment could lead to variation in the local microbiota. In particular, the dynamic alternation of the lung microbial structure in patients with chronic lung diseases, such as COPD, asthma, cystic fibrosis, and idiopathic pulmonary fibrosis, has a significant association with disease exacerbations (Dickson et al., 2014; O'Dwyer et al., 2016).

Several sputum-based microbiota analyses demonstrate that TB patients exhibit altered community diversity compared to healthy subjects, and a number of distinct foreign bacteria have been identified in the sputum samples from TB patients (Cui et al., 2012; Wu et al., 2013; Krishna et al., 2016). In those studies, microbes such as *Streptococcus* and *Pseudomonas* are dominant in TB samples, yet there is no clear consensus on the frequency and abundance of the bacterial genera defined in TB patients. It should be pointed out that there is actually a triad interaction in TB patients, which encompasses *M. tuberculosis*, indigenous microbiota, and the host. For instance, a recent study demonstrates that the native microbiota are able to enhance IL-17A production in the lung and stimulate alveolar macrophages in response to respiratory infection (Brown et al., 2017).

Another important issue relating to the intricate regulation of TB infection and the microbiome is the reciprocal connection of the gut and lung, which is known as the gut-lung axis. A number of recent studies indicate that in a given circumstance, the gut microbiota either protect the lung from infectious diseases such as pneumococcal pneumonia and TB (Khan et al., 2016; Schuijt et al., 2016) or aggravate the lung autoimmunity because of excessive Th17 responses (Bradley et al., 2017). More studies are required to fully understand the links between *M. tuberculosis* infection and the microbiota within the gut-lung axis. Results from such studies may help in the development of rational diets and antibiotic therapies for TB patients.

## Conclusion

*M. tuberculosis* is the most successful pathogen that adapts to survive within host intracellular microenvironments. Rather than rashly inducing acute inflammation, it takes “compromised” countermeasures to enter into a quiescent latency status to elude host immune clearance. During the lengthy latency period, *M. tuberculosis* employs a range of effector proteins to reinforce its living niches and counterbalance host immune defenses. Although latent TB infection can last for decades without exacerbation, the infected individuals are likely enduring persistent impacts of *M. tuberculosis*, resulting in metabolic disturbance, immune imbalance, and microbiota dysbiosis. We summarized typical human diseases frequently occurring in TB patients (Table 2). However, many questions remain unresolved. Which immune pathways are involved in preventing the latent mycobacteria from being reactivated and determining the progress of granulomas? How could the dormant mycobacteria sense the changes within the host microenvironment to start replicating and causing complications? Is *M. tuberculosis* infection an initial cause or an accompanying outcome of metabolic syndromes in patients? Which biomarkers could be used for distinguishing TB from other non-infectious diseases?

**Table 2:** Examples of studies on mechanisms underlying the interaction between TB infection and multiple human diseases.

**Disease**	**Correlation to TB**	**Relevant findings**	**References**
**PULMONARY DISEASE**			
Pneumonia	TB infection as a potential etiology	TB infection increases susceptibility to secondary bacterial pneumonia in young children	Oliwa et al., 2015a
COPD ^*^	Increases the risk of active TB; TB infection as a potential etiology;	TB infection leads to remodeling of the lung architecture, such as extensive fibrosis, cavitation, traction bronchiectasis, bronchostenosis, or parenchymal lung destruction; the development of bronchiectasis in patients with COPD causes active TB	Dheda et al., 2005; Chakrabarti et al., 2007; Jordan et al., 2010
Lung cancer	TB infection as a potential etiology	TB infection establishes chronic and persistent inflammation; induces production of NO and ROS to bring about DNA damage; develops pulmonary fibrosis	Ardies, 2003; Sharma et al., 2004; Shin et al., 2008
**AUTOIMMUNE DISEASE**			
Sarcoidosis	TB infection as a potential etiology	*M. tuberculosis* HSP16 and HSP70 participate in the etiopathogenesis of sarcoidosis; activation of TLRs signaling caused by *M. tuberculosis* infection involves in the pathogenesis of pulmonary sarcoidosis	Dubaniewicz et al., 2006; Gabrilovich et al., 2013
SLE ^*^	Increases the risk of active TB; TB infection as a potential etiology;	Cross-reactivity between mycobacterial and host self-antigens; antigenic resemblance between mycobacterial glycolipids and host DNA; immune abnormalities and immunosuppressive therapy lead to active TB development	Amital-Teplizki et al., 1989; Yun et al., 2002
**METABOLIC DISEASE**			
DM ^*^	Increases the risk of active TB; promotes TB progression	Promotes mycobacterial proliferation; enhances CD4^+^ Th1/Th17 responses and reduces frequencies of Treg cells in active TB patients; reduces Th1/Th17 responses in latent TB patients	Martens et al., 2007; Kumar et al., 2013, 2014
Obesity	Decreases the risk of active TB	The adipose tissue may have immunomodulatory functions against TB infection; the mechanisms are still largely unknown	Wieland et al., 2005; Ordway et al., 2008; Abella et al., 2017
Atherosclerosis	TB infection as a potential etiology	*M. tuberculosis* HSP65 accelerates the progression of atherosclerosis; both of two diseases accumulates foamy macrophages in the lesion	Peyron et al., 2008; Zhang et al., 2012; Chinetti-Gbaguidi et al., 2015; Wick et al., 2017
Hypovitaminosis D	TB infection as a potential etiology	Vitamin D is essential for production of antimicrobial peptide and promotion of autophagy and phagosomal maturation; the mechanisms of *M. tuberculosis*-induced hypovitaminosis D is still unclear	Liu et al., 2006; Campbell and Spector, 2012; Azam et al., 2016; Wang J. et al., 2017
**CO-INFECTION**			
HIV-1	Increases the risk of active TB; TB infection results in increased viral replication	Depletes *M. tuberculosis*-reactive T cells; inhibits phagocytosis and autophagy; induces cell death and tissue necrosis	Bell and Noursadeghi, 2017
*Helicobacter pylori*	Decreases the risk of active TB	Enhances host Th1-type responses with higher level of IFN-γ, IL-2, TNF-α, and CXCL-10	Perry et al., 2010
Helminth	Commonly occurs in TB patients; disturbs host immune responses to either of infectious pathogens	Reduces *M. tuberculosis*-antigen specific immune responses; lowers Th1/Th17 responses and elevates Th2 responses	Babu and Nutman, 2016

* *COPD, chronic obstructive pulmonary disease; SLE, systemic lupus erythematosus; DM, diabetes mellitus*.

The adaptable features of *M. tuberculosis* also suggest for more rational combinatory treatments for TB patients. According to the latest standard regimen, drug-susceptible TB requires a 6-month therapy with a combination of rifampicin, isoniazid, pyrazinamide, and ethambutol, while RR/MDR-TB treatment can last up to 12 months (WHO, 2017). The excessively long course of treatment with those antibiotics inevitably causes multiple adverse effects, such as pulmonary impairment, hepatotoxicity, hypothyroidism, electrolyte abnormalities, and visual and hearing disturbance (Horsburgh et al., 2015). The multicomponent Chinese herbal medicines could have synergistic anti-TB effects by regulating the immune system and keeping it at a balanced status, which is frequently skewed during chronic inflammation and infectious diseases (Pan et al., 2011; Posadzki et al., 2013; Gupta et al., 2017). Nevertheless, the molecular details underlying the mutual regulation of *M. tuberculosis* and multiple comorbidities are far from being fully understood. More in-depth and delicate studies are warranted to better understand the intricate relationships and dynamic regulation among *M. tuberculosis*, host immunity, and the host microbiota, both in physiological and under pathological conditions. Such knowledge is critical for developing rational treatments to better control TB and to help alleviate its associated comorbidities.

## Author contributions

QC and YZ wrote a preliminary draft of this manuscript. CL directed this work and revised the manuscript draft. All authors reviewed and approved the manuscript.

### Conflict of interest statement

The authors declare that the research was conducted in the absence of any commercial or financial relationships that could be construed as a potential conflict of interest.
